# Comparison of Dechlorane Plus Concentrations in Sequential Blood Samples of Pregnant Women in Taizhou, China

**DOI:** 10.3390/molecules27072242

**Published:** 2022-03-30

**Authors:** Ji-Fang-Tong Li, Xing-Hong Li, Yao-Yuan Wan, Yuan-Yuan Li, Zhan-Fen Qin

**Affiliations:** 1 State Key Laboratory of Environmental Chemistry and Ecotoxicology, Research Center for Eco-Environmental Sciences, Chinese Academy of Sciences, 18 Shuangqing Road, Haidian District, Beijing 100085, China; m17853529621@163.com (J.-F.-T.L.); wanyaoyuan21@mails.ucas.ac.cn (Y.-Y.W.); yyli@rcees.ac.cn (Y.-Y.L.); qinzhanfen@rcees.ac.cn (Z.-F.Q.); 2 University of Chinese Academy of Sciences, 19A Yuquan Road, Shijingshan District, Beijing 100049, China

**Keywords:** dechlorane plus, maternal blood, sequential samples, variation, correlation

## Abstract

To develop an appropriate sampling strategy to assess the intrauterine exposure to dechlorane plus (DP), we investigated DP levels in sequential maternal blood samples collected in three trimesters of pregnancy, respectively, from women living in Taizhou. The median concentration of DPs (sum of *syn*-DP and *anti*-DP) in all samples was 30.5 pg g^−1^ wet-weight and 5.01 ng g^−1^ lipid-adjusted weight, respectively. The trimester-related DP concentrations were consistently strongly correlated (*p* < 0.01), indicating that a single measurement of DP levels could represent intrauterine exposure without sampling from the same female repeatedly; however, the wet-weight levels significantly increased across trimesters (*p* < 0.05), while the lipid-adjusted levels did not significantly vary. Notably, whether lipid-adjusted weight or wet-weight levels, the variation extent of DP across trimesters was found to be less than 41%, and those for other persistent organic pollutants (POPs) reported in the literature were also limited to 100%. The limitation in variation extents indicated that, regardless of the time of blood collection during pregnancy and how the levels were expressed, a single measurement could be extended to screen for exposure risk if necessary. Our study provides different strategies for sampling the maternal blood to serve the requirement for assessment of in utero exposure to DP.

## 1. Introduction

Dechlorane plus (DP) is a high-chlorinated organic flame retardant. For decades, various products containing DP have been widely used in the electric/electrical industry, such as commercial wire and cable, and connectors for computers and televisions. [1]. Since DP is considered an alternative to decabromodiphenyl ether (BDE-209), which has been phased out [2,3], its application may be expanded in the future. DP exhibits some POP-like properties, one of which is its ability to accumulate in animals and humans [2,4,5]. Studies on animals [6,7] (e.g., fish and poultry) have given evidence on DP transfer from parents to offspring, and Ben’s study [8] further confirmed the transplacental transfer of DP in humans. Although its health risk to humans has not yet been better understood until now, animal experiments have shown that exposure to DP in early life could impact axonal growth, musculature, and motor behavior in embryo–larval zebrafish [9], and regulate mRNA expression in chicken embryos [10]. Due to the significant linkage between prenatal exposure and adverse health outcomes at birth and/or in later life, exposure of pregnant women to persistent organic contaminants is an ongoing public health concern [11]. The transplacental transfer and potential toxicity to animals [6,7], together with POP-like properties [2,4,5], implies that we ought to pay specific concern to in utero exposure to DP in humans.

Epidemiological studies generally used a single measurement of maternal blood samples to assess in utero exposure to persistent chemicals [12]. This strategy was cost-efficient, easy to collect and store samples, and also reduced the burden on participants in large epidemiological studies [12,13]; however, by measuring the levels of persistent pollutants in sequential maternal blood samples, some studies had shown that levels of certain POPs might change during pregnancy [14,15,16,17,18]. This was not surprising since important physiological changes would occur over the course of pregnancy [19,20]. Accompanied by these physiological changes, some internal POPs may be redistributed among human compartments, further leading to a change in maternal blood concentrations across gestation [13,18,21,22]. In addition, it was found that changing patterns of levels (based on wet-weight and/or lipid-adjusted weight) in sera of pregnant women for these POPs might differ from compound to compound. For example, the wet-weight levels of per- and polyfluorinated compounds (PFCs) [16,17,18] showed a general decrease over the course of pregnancy, while those of polychlorinated biphenyls (PCBs)/organic chlorinated pesticides (OCPs) showed a general increase [15,23,24]. In any case, doubts have been raised about the feasibility of using a single measurement to represent in utero exposure to POPs.

The maternal serum that could be a proxy of cord serum for assessing in utero exposure to DP has been documented [8]; however, DP showed different tissue distribution patterns in humans from lipid-related POPs (e.g., PCBs and PBDEs) [8,25,26,27] and protein-binding PFCs [28,29]. Pan et al. [26] believed that the specific distribution patterns of DP in humans might be regulated together by both lipids and non-lipid factors in the circulatory system. As a result, the prior experiences from POPs reported (e.g., PCBs and PFCs) on targeting one collection of maternal blood during pregnancy to assess in utero exposure [30,31] might not apply to DP. In order to develop an appropriate sampling strategy to obtain knowledge of in utero exposure to DP, we recognized the need to investigate DP levels in different time windows during gestation.

In this study, we determined DP levels in sequential maternal blood samples taken during three trimesters of pregnancy. The main purpose was to examine the inter-period correlations and to investigate the variation patterns/extent of DP concentrations during pregnancy. To the best of our knowledge, this is the first thorough examination of variation in maternal DP levels during different stages of pregnancy.

## 2. Materials and Methods

### 2.1. Sample Collection

Since the 1970s, many disposal e-waste products have been transported to Taizhou for recycling purposes. These old devices were generally recycled in informal family-run workshops with primitive methods, such as burning piles of wires and melting circuit boards over coal grills, which allowed the complex chemicals to be easily transferred to the surrounding environment. Consequently, the Taizhou region had serious environmental problems and local residents were at high health risk of exposure to various e-waste-related contaminants, including DP [8,32]. This study was undertaken from 2016 to 2017 in Taizhou, China. Pregnant women living in the Taizhou area with higher exposure levels of DP [25,26,27] were an ideal group to study fetal health risks associated with DP; therefore, we recruited forty pregnant women in this region as our study participants, who agreed to provide us with serum samples leftover from gestational routine blood monitoring. All pregnant women were residents living near e-waste recycling sites, but they were not engaged in e-waste recycling work. All the donors signed informed consent forms and provided basic personal information in the form of a questionnaire at the first pregnancy medical examination. Demographic characteristics of the participants included age (mean: 27 years; range 19–35 years), pre-pregnancy body mass index (BMI, mean: 21.2 kg m^−2^; range: 17.6–28.2 kg m^−2^), and parities (20 primiparas and 6 multiparas). The sampling time of maternal blood was arranged at three trimesters, which were classified as follows: the first trimester, <14 weeks; the second trimester, 14 to 28 weeks; the third trimester, >28 weeks. Blood samples from the 40 women who completed the study might not have all been obtained across three trimesters of pregnancy. As long as two trimesters samples could be taken from the same pregnant woman, the sample data of this woman was included in the study statistics. Finally, a total of 75 samples were obtained, including 26 in the first trimester, 25 in the second trimester, and 24 in the third trimester. A valid sample pair consisted of at least two sequential samples taken in different trimesters. Finally, we obtained 21 pairs between 1st and 2nd trimesters, 20 pairs between 1st and 3rd trimesters, and 19 pairs between 2nd and 3rd trimesters; the valid paired samples were from 30 participants. The average time interval (range) of samples from the same participant taken was 88 days (range: 48–126 days) between the 1st and 2nd trimester, 63 days (range: 35–118 days) between the 2nd and 3rd trimester, and 147 days (range: 91–196 days) between 1st and 3rd trimester, respectively (Table S1). We commissioned hospital staff responsible for collecting these trimester-dependent matched samples during pregnancy. The institutional review board of the Research Center for Eco-Environmental Sciences and the First People’s Hospital of Wenling approved the study protocol prior to the collection of samples.

### 2.2. Sample Pretreatment and Chemical Analysis

The extraction and purification of samples were mainly carried out according to the procedures reported by Ben et al. [25]. In brief, the serum sample was spiked with ^13^C_10_-labelled *syn*-DP and ^13^C_10_-labelled *anti*-DP (4 ng) as surrogate standards, then denatured with hydrochloric acid and isopropanol, and ultrasonically extracted with a mixture of methyl tert-butyl ether and hexane with three times repeats. The extracts were purified by a multi-layer chromatography column, and were washed by a mixture of hexane and dichloromethane. The eluent was concentrated to 20 μL for further analysis, and ^13^C_12_-labelled CB-208 as the injection standard was added before injection.

The target compounds were determined by Agilent 6890 gas chromatography coupled (Agilent Technologies Inc, Santa Clara, CA, USA) with 5973 low-resolution mass spectrometry (Agilent Technologies Inc, Santa Clara, CA, USA) in negative chemical ionization mode. The detailed information on chromatographic separation and the monitored ion fragments of target chemicals could be found in the reports of Ben et al. [25,28]. The method detection limits (MDLs) were defined as three times the standard deviation (SD) of the concentration (4 pg g^−1^) of the target compounds spiked into matrix blank samples (bovine serum). MDLs were 0.8 pg g^−1^ ww and 0.14 ng g^−1^ lw for *syn*-DP, respectively. MDLs were 0.7 pg g^−1^ ww and 0.12 ng g^−1^ lw for *anti*-DP, respectively. The recoveries of surrogate standards were 73–111% for ^13^C_10_-*syn*-DP and 65–108% for ^13^C_10_-*anti*-DP. The solvent blank, matrix blank, and procedural blank were performed with each batch of samples, and no interferences were found in these quality control samples.

### 2.3. Serum Lipid Contents and Blood Biochemical Parameters

Serum lipid contents could be obtained generally by two assays. One was calculated according to blood lipid biochemical parameters (e.g., total cholesterol and total triglyceride) [21], and the other was directly determined by the gravimetric method [33]. In the present study, as TC and TG values in some blood samples were not available, a gravimetric method was used to obtain the information for all participants. Levels of blood biochemical parameters were obtained from the local hospital.

### 2.4. Data Analysis

All analyses were conducted using SPSS 23.0 (SPSS Inc., Chicago, IL, USA). The Kolmogorov–Smirnov test was used to test the normality of the distribution of continuous variables. The paired-sample *t*-test was used to compare the difference between log-transformed inter-trimester levels of target compounds, as well as inter-trimester levels of serum lipid contents and blood biochemical parameters. One-sample *t*-test was used to compare the difference in blood lipid contents between data from our study and Barr’s study [12]. Pearson correlation analysis was employed to examine the relationships among log-transformed inter-period concentrations. Partial correlation analysis was further used to examine the influence of age and sampling day intervals on the correlation. *p*-values < 0.05 were considered statistically significant.

The change in serum concentrations and serum lipid contents were evaluated from paired observations by trimesters. The change ($∆C_{i})$ were defined as the difference in concentration between the later sample and the matched earlier sample, according to Equation (1). In Equation (1), $C_{it}$ represents value measured in a sample taken in trimester t (where *t* = 2 or 3) for the $i^{th}$ participant. $C_{it^{\prime }}$ represents value measured in a sample taken in trimester $t^{,}$ (where when *t* = 2, $t^{\prime }$ = 1; and when *t* = 3, $t^{\prime }$ = 2 or 1) for the $i^{th}$ participant.

(1) $$∆C_{i}\;=\;C_{it}\;-\;C_{it^{\prime }}$$

The variation extent (P, %) was defined as $C_{i}$ divided by $C_{it\prime }$, then multiplied by 100, as expressed in Equation (2).

(2) $$P\;=\;100\;\times \;C_{i}/C_{it^{\prime }}$$

## 3. Results

### 3.1. DP Concentrations in All Samples

Table 1 listed DP levels in all samples throughout pregnancy, based on wet-weight (ww) and lipid-adjusted weight (lw). It was found that DP could be detected in all samples, and the whole data set showed abnormal distribution. DPs in Table 1 referred to the sum of *syn*-DP and *anti*-DP, and the median level was 5.01 ng g^−1^ lw and 30.5 pg g^−1^ ww, respectively.

Pan et al. [26] summairzed the DP body burden (median or mean value) in humans from different countries or regions. It was found that the DP levels (Table 1) in this study were within the range of those of the general population living near the contamination sources, such as e-waste recycling areas [25,27,34], municipal solid waste incinerators [35], and previous DP production areas [36]. The levels were higher than those of the general population [35,37,38,39,40,41]. This was consistent with the fact that e-waste recycling activities were an important source of DP for human exposure [35,37,38,39,40,41]. Moreover, the comparison also suggested that pregnant Chinese women living in this area had a relatively high body burden of DP. In addition, the highest levels of DP were found to be 136 ng g^−1^ lw in this study, but Ben et al. reported that DP levels could be up to 900 ng g^−1^ lw in sera of pregnant women and 89.7 ng g^−1^ lw in cord serum [25], and 590 ng g^−1^ lw in breast milk [25]. In addition, placental transfer of DP in humans had been confirmed and DP concentration in the cord serum was estimated to be 38% of that in the maternal serum [8]. These results demonstrated that the bioaccumulation of DP could reach very high levels in pregnant women and their fetuses, urging the need to assess the health risk of pregnant women and their fetuses/infants exposed to DP. Pregnant women and fetuses are sensitive to chemical exposure, so these elevated levels indicated the need to assess the health risk of pregnant women and their fetuses/infants exposed to DP.

### 3.2. Trimester-Related Characteristics of DP Concentrations

#### 3.2.1. Trimester-Related Concentrations

**Figure 1:**
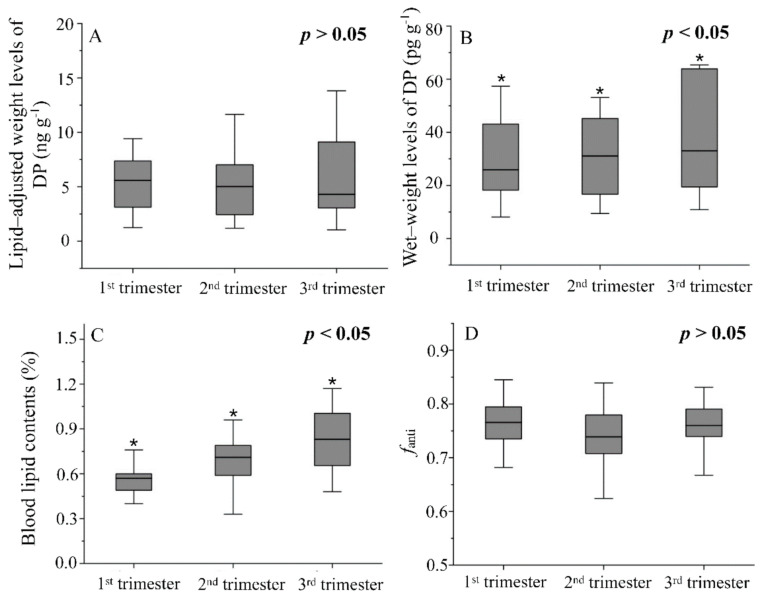
Box plot (minimum, 25% quartile, median, 75% quartile, and maximum; outliers were not shown) of lipid-adjusted levels of DP (**A**), wet-weight levels of DP (**B**), blood lipid contents (**C**), and *f*_anti_ (**D**) of samples during three trimesters. (* trimester-related levels showed significant differences at *p* < 0.05. The sample sizes for the first, second, and third trimesters were 26, 25, and 24, respectively).

The Kolmogorov–Smirnov test showed that the data set for trimester-related DP levels (both ww and lw) did not conform to a normal distribution (*p* < 0.000). The median level of DP in serum was 25.9 pg·g^−1^ ww in the 1st trimester, 31.1 pg·g^−1^ ww in the 2nd trimester, and 33.0 pg·g^−1^ ww in the 3rd trimester of pregnancy, respectively (Figure 1B). After lipid adjustment, the median level was 5.59 ng·g^−1^ in the 1st trimester, 5.01 ng·g^−1^ in the 2nd trimester, and 4.30 ng·g^−1^ in the 3rd trimester, respectively (Figure 1A).

#### 3.2.2. Inter-Trimester Associations

A strong association was found between inter-trimester DP levels, whether wet-weight basis or lipid-adjusted weight basis (*p* < 0.01, r > 0.675), as shown in Table 2. For the two isomers, similar results could be found (*p* < 0.01). Age was believed to be an important determinant of the body burden of persistent organic pollutants [22,42,43]. Moreover, day intervals between two sequential samples were also perceived as an important factor affecting the inter-period correlation coefficient [13,16]; however, our results of Partial correlation analysis indicated that the correlations among trimesters still remained significant (*p* < 0.01) (Table 2). Similar, significant inter-trimester correlations were also reported for other POPs in most of the published literature (e.g., PCBs, OCPs, and PFCs), as summarized in Table S3. It seemed to be a general rule that POP levels in sequential maternal blood samples had a significant inter-trimester correlation.

#### 3.2.3. Variation Patterns

Box plots in Figure 1A,B displayed the variation patterns of DP levels (logarithm transformation) based on lipid-adjusted weight and wet weight, respectively. Based on wet-weight form (Figure 1B), levels of DPs showed a small but significant increase as the pregnancy continued (*p* < 0.05). Specifically, the levels in the 2nd and 3rd trimesters were significantly higher than those in the 1st trimester (*p* = 0.009 and 0.002, respectively, paired-samples *t*-test), and the levels in the 3rd trimester were also significantly higher than those in the 2nd trimester (*p* = 0.040, paired-samples *t*-test); however, for lipid-adjusted levels (Figure 1A), the median value was on a downward trend from the 1st to 3rd trimester, but significant differences were not found for log-transformed levels (*p* > 0.05, paired-samples *t*-test). The results indicated that lipid-adjusted levels of DPs showed a different variation pattern from that of the wet-weight levels in sequential blood samples from pregnant women.

#### 3.2.4. Implications Based on Associations and Variation Patterns

Both inter-trimester associations and variation patterns of DP levels gave two implications about sampling strategies for in utero exposure to DP. The first one was related to the frequency of blood samples taken from the same individual participant, and the other was related to the time window for collecting a sample for different participants.

First, the strong inter-trimester associations of DP levels (both wet weight and lipid-adjusted weight) observed in our study suggested that blood samples taken at one trimester of pregnancy could act as a proxy for the other two trimesters when assessing in utero exposure to DP. It was sufficient to make one single measurement of levels in sera taken within a given time window. Then, the insignificant variation of lipid-adjusted DP levels across trimesters suggested that regardless of the sampling time windows, a single sample measurement based on lipid-adjusted levels could reflect DP exposure throughout gestation; however, for assessment based on wet-weight levels, the sampling time window was required to be within a narrow range because of the significant difference of wet-weight levels by trimesters.

In any case, it should be kept in mind that when a single measurement was expected to reflect DP exposure throughout gestation, the sampling time window based on wet-weight levels should be different from that based on lipid-adjusted weight levels. It was more flexible and practical to use lipid-adjusted DP levels to assess in utero exposure to DP due to the unlimited collection time of blood samples.

### 3.3. Blood Biochemical Parameters and Effect on Variation of DP Levels

#### 3.3.1. Blood Parameters and Trimester-Related Characteristics

Biochemical parameters of maternal blood provided by the local hospital, including total cholesterol (TC), total triglyceride (TG), high-density lipoprotein (HDL), low-density lipoprotein (LDL), apolipoprotein A (Apo-A), apolipoprotein B (Apo-B), lipoprotein a [Lp(a)], hemoglobin (Hb), albumin (ALB), and creatinine (Cre) are listed in Table S2. These listed parameters reflected the lipid/protein profiles and blood volume of the pregnant women. The average values of the lipid-related parameters, including TC, TG, Apo-A, Apo-B, and Lp(a), were above or at the top of the reference intervals, while those for Hb, ALB, and Cre, were on the bottom of the reference intervals. The relatively higher lipid-related parameters in pregnant women are believed to be related to the need for more energy supply for mothers and fetuses during pregnancy [14,19,21]. The observed decreases in maternal serum Hb, ALB, and Cre might be associated with pregnancy-related increases in body and blood volume [18,44,45]. Table S2 shows the trimester-related data of these biochemical parameters. TC, TG, and Apo-B significantly increased (paired-sample *t*-test, *p* < 0.05). In contrast, the non-lipid parameters (e.g., Hb and ALB) showed a significantly decreasing trend (paired-sample *t*-test, *p* < 0.05). The results supported the occurrence of maternal physiological changes by trimesters, and distinct variation patterns for lipid-related and non-lipid biochemical parameters. Because of the small paired-sample size, data from the 2nd trimester were not included for comparison.

The data of lipid contents conformed to normal distribution. As a whole, the value varied from 0.330% to 1.17%, with 0.691 as the mean value across trimesters (Table 1). The average value (0.691%) was significantly higher (*p* < 0.000, one-sample *t*-test) than that in the general population (0.5–0.6%) [12]. Then, the average value in the 1st, 2nd, and 3rd trimesters was 0.567%, 0.696%, and 0.820%, respectively. As shown in Figure 1C, trimester-related lipid contents increased gradually and showed significant differences between each other by paired-sample *t*-test (*p* = 0.001 between the 1st and 2nd trimester, *p* < 0.001 between the 1st and 3rd trimester, and *p* = 0.018 between the 2nd and 3rd trimester, respectively). The increasing tendency of lipid contents during pregnancy was consistent with practical experiences and the reports from other published literature [12].

In short, since the distribution and redistribution of DP in humans might be driven by both blood lipids and proteins [26,27], the trimester-related variation of blood parameters in the circulatory system implied the possible change of DP levels as pregnancy proceeded.

#### 3.3.2. Effect of Blood Biochemistry Parameters on Variation of DP Levels

DP showed certain specific distribution behaviors among human tissues with the preferential accumulation in the blood compared to adipose tissue, placental, and breast milk [8,27], implying the potential strong interaction with blood lipids and non-lipids compositions. Importantly, it had been suspected that both lipid and non-lipid factors in the bloodstream might exert an important impact on the variation of POP levels during pregnancy [18,24]. Our study found that lipid-adjusted DP levels by trimesters were very close to each other (Figure 1A) (*p* > 0.05), while the wet-weight levels increased in parallel with the increase in lipid contents during pregnancy (Figure 1B,C). The stable lipid-adjusted DP levels across pregnancy indicated that lipid content could almost completely correct the differences in inter-trimester DP wet-weight levels; therefore, change in blood lipid contents during pregnancy was the major factor resulting in a change in wet-weight DP levels. Adipose tissue was considered the main sink of DP in humans [26]. The mobilization of store lipids during pregnancy with concomitant redistribution of DP from adipose tissue to the circulatory system might increase serum wet-weight DP concentrations as pregnancy gestation. Hansen et al. [14] found a similar phenomenon in organochlorines (OCs), with wet-weight levels and lipid contents peaking at birth, then this peak disappeared when OC concentrations were adjusted by lipid contents. Hansen et al. [14] believed that the wet-weight OC levels during gestation might be driven by physiological lipids.

**Figure 2:**
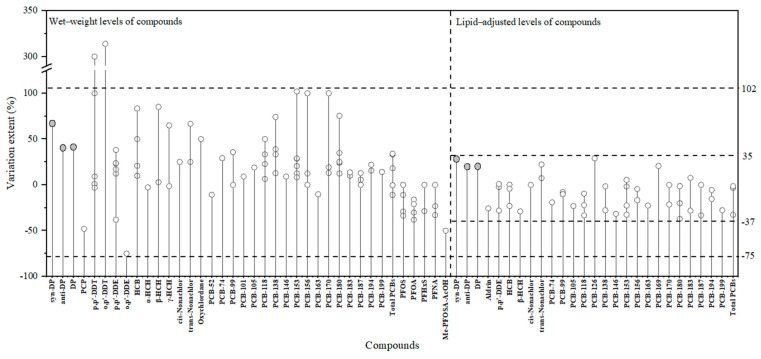
Variation extent (%) of DP in this study and other POPs in published literature. (The grey “○” represents the variation extent of DP in this study, and the blank “○” represents the variation extent of POPs in published literature, detailed information is available in Table S3. The left part of the dotted line represents the levels of DP and POPs based on wet weight, and the right part of the dotted line represents the levels of DP and POPs based on lipid-adjusted weight. The horizontal dotted lines represent the range of POP variation extents based on wet-weight and lipid-adjusted levels.).

In addition, we found a distinct difference in the various patterns of the wet-weight levels between PFCs and PCBs/OCPs, as summarized in Figure 2. The wet-weight serum levels of PFCs usually showed a trimester-related decrease [16,17,18], while the wet-weight levels of PCBs/OCPs usually showed a trimester-related increase [15,23,24]. It was well known that PFCs were apt to bind to serum albumin [28,29], whereas PCBs/OCPs showed a strong dependence on blood lipids [14]. With the progress of pregnancy, the decreased albumin levels and increase in blood volume might reduce the redistribution of PFCs in the bloodstream, resulting in decreasing wet-weight levels [16]. In contrast, the increasing lipid contents during pregnancy might cause the elevated redistribution of PCBs/OCPs in maternal blood, leading to an increase in serum wet-weight levels [14]. DP showed a similar variation pattern with PCBs/OCPs rather than PFCs, likely ascribing to its more similarity in lipophilicity with PCBs/OCPs. Although the interaction of DP with certain proteins in the bloodstream (e.g., serum lipoproteins and albumin) might play an important role in its distribution in humans [26,27], the lipid-adjusted DP levels by trimesters did not significantly differ from each other, implying that non-lipid factors in blood circulatory system might not dominantly affect the variation patterns of DP levels.

In short, the results demonstrated that changes in DP levels observed during pregnancy were associated with variation in blood lipids to a great extent.

### 3.4. Stereo-Selective Profile of DP during Pregnancy

The stereo-selective profile of two DP isomers was examined by the fraction of anti-DP (anti-DP/∑DP, *f*_anti_). In the present study, *f*_anti_ values were not normally distributed, so that paired samples t-test was used to examine the difference in *f*_anti_ values between trimesters. As shown in Table 2, the median value of *f*_anti_ in all sera was 0.751, which was within the data range for the general population [25]. Furthermore, the median value in the 1st, 2nd, and 3rd trimester was 0.758, 0.744, and 0.760, respectively. No significant difference among the three gestational stages was found (*p* > 0.05) (Figure 1D), suggesting that stereo-selective DP bioaccumulation in maternal blood did not occur as a result of pregnancy. Note that the stereoselective bioaccumulation of DP isomers during transport from mother to fetus has been documented [25]. These suggested that both maternal physiological changes during pregnancy and the occurrence of transplacental transport of DP were not enough to influence the status of its stereoselective bioaccumulation in maternal blood.

### 3.5. Variation Extents in Levels of DP and Other POPs across Gestation

#### 3.5.1. Variation Extent of DP

The variation extent of levels in sequential blood specimens for specific POPs was helpful to outline the fluctuation power of levels across pregnancy. The variation extent of trimester-related DP levels was calculated based on Equations (1) and (2) and the results are displayed in Figure 2. As a result, the median variation extent of wet-weight levels was 30% between the 1st and 2nd trimester, 41% between the 1st and 3rd trimester, and 15% between the 2nd and 3rd trimester, respectively. Based on lipid-adjusted levels, the median value of variation extent was −13%, 20%, and 6%, respectively. Obviously, variation extents based on lipid-adjusted DP levels were less than those based on wet-weight levels, further confirming the effect of blood lipids on DP levels across gestation. In short, it was found that DP levels across trimesters of pregnancy varied by no more than 41% (Figure 2), whether based on wet-weight or lipid-adjusted weight.

#### 3.5.2. Variation Extent of Other POPs

There have been a dozen studies to investigate the variation of POP levels in sequential maternal blood samples across pregnancy [13,14,15,16,17,18,21,22,23,24,37,46,47,48,49]. Unfortunately, most published literature focused on the level differences (significant or insignificant) and/or variation patterns (increasing, decreasing, or stable), discussion on the variation extent in POP levels throughout pregnancy was restricted [16,21,23]. To better understand the variation details across pregnancy, we calculated the variation extent of POP levels reported in the published literature according to Equations (1) and (2); however, since only statistical descriptions of POP levels (e.g., mean value, median value, or geometric mean value) were available in the published literature, the statistical values (detailed information available in Table S3), were used to estimate the variation extents. In order to reflect the maximum variation extents of POP levels across pregnancy, the variation extents listed in Figure 2 were calculated by levels from the sequential serum samples with the longest sampling time intervals in the literature. For example, Adetona et al. [23] collected three batches of matched blood samples in the first, second, and third trimesters of pregnancy. The values presented in Figure 2 were data only related to the 1st and 3rd trimesters.

Figure 2 shows that the variation extents based on the lipid-adjusted levels of POPs were negatively limited to 37% and positively limited to 35%, respectively. Variation extents based on wet-weight levels were limited to 75% negatively and 102% positively in all but two studies [48,50]. The largest variation extent of the DDT levels, about 300% as reported by Tasker et al. [48], might be due to the concentrations close to its limitation of detection. In the report of Curley et al. [50], sequential maternal blood samples were taken from only five females, and the levels of both o,p’-DDT and o,p’-DDE, varied widely among individual donors. Nevertheless, Curley et al. only reported the mean value of levels in the same period; the data listed in Figure 2 were calculated by the mean value and possibly contributed to the large variation extents.

Clearly, regardless of wet-weight basis or lipid-weight basis, the variation extent of the POP levels did not exceed 100% for almost all individual compounds reported in the literature.

#### 3.5.3. Implication from Variation Extents

The limited variation extent found in Figure 2 (Table S3) indicated a limited misclassification risk for POP exposure when a single measurement without information on the sampling time window of samples was used. As a result, this observation could inform the appropriate application of sampling strategy to assess in utero exposure to POPs in certain cases. For example, the sampling strategy might be a single measurement without specific sampling time windows for screening purposes. Notably, the reported compounds were limited to PCBs, OCPs, and PFCs in the literature and DP in this study. In addition, the variation extent from the published literature was calculated by statistical values, not by the inter-trimester levels of each participant. Additional studies are needed to insightfully investigate the variation patterns and variation extent of more POPs to generalize this principle. 

### 3.6. Limitations

Although the sampling period covered three trimesters of pregnancy in our study, the days of sampling time intervals between the 1st and 3rd trimester was only 147 (35–196) days (about half of the gestational period). As a result, the relatively short time intervals in this study might restrict our understanding of the full information on the variation in DP levels across the whole pregnancy period (10-month intervals or more). In addition, in terms of the results in Figure 2, it was found that the homologous group of compounds or even the same individual compounds did not subject to uniform variation patterns of levels in different literature [14,15,16,17,18,22,23,24,37]. These differences might be ascribed to maternal weight gain [20,21], the time since peak exposure (past, recent, and current) [14], as well as baseline concentrations [13,22]. Unfortunately, no information on these factors was available in this study, limiting further discussion of their effect on levels during pregnancy.

## 4. Conclusions

In summary, a single measurement could be used to assess in utero exposure to DP, whether based on wet-weight levels or lipid-adjusted levels. The accurate evaluation results based on the lipid-adjusted levels could be obtained regardless of the sampling time window of serum samples; however, the assessment results based on wet-weight levels were reliable only when the serum sampling time was limited to a narrow window. Furthermore, the limited variation of period-related levels was found for DP and other POPs, suggesting that it is feasible to use a sample of pregnant women to roughly evaluate in utero exposure risk, regardless of the sampling time window and how the levels were expressed (lw or ww). Stereoselective behaviors of DP isomers in maternal sera did not occur during pregnancy. In conclusion, these results will serve to design an appropriate sampling strategy for assessing in utero exposure to DP. Additional factors need to be considered in future studies, including the time span throughout gestation for sequential blood samples and full information on physiological changes during pregnancy.

## Figures and Tables

**Table 1:** Descriptive statistics for dechlorane plus (DP) and serum lipid contents measured in all serum samples of pregnant women.

Compound	All Serum Samples				
	*N*	Median	Mean	SD	Range
Wet-weight (pg g^−1^)					
*syn*-DP	75	7.56	17.7	33.1	1.26–199
*anti*-DP	75	21.4	54.6	109	6.76–602
∑DPs	75	30.5	72.3	142	8.12–801
Lipid-adjusted weight (ng g^−1^)					
*syn*-DP	75	1.10	2.70	5.20	0.190–33.9
*anti*-DP	75	3.45	8.15	16.5	0.740–102
∑DPs	75	5.01	10.9	21.6	1.04–136
*f*_anti_	75	0.751	0.746	0.0653	0.440–0.845
Lipid contents(%)	75	0.670	0.691	0.192	0.330–1.17

*N*: The number of all serum samples in our study. SD: Standard deviation; Range: minimum to maximum.

**Table 2:** Unadjusted/adjusted inter-period correlation of log-transformed trimester-related DP levels.

	Unadjusted				Adjusted Covariate ^a^		
	1st–2nd	1st–3rd	2nd–3rd		1st–2nd	1st–3rd	2nd–3rd
Wet-weight (pg g^−1^) ^b^							
*syn*-DP	0.892	0.785	0.935		0.786	0.675	0.762
*anti*-DP	0.928	0.862	0.902		0.861	0.791	0.763
∑DPs	0.930	0.859	0.908		0.864	0.831	0.774
Lipid-adjusted weight (ng g^−1^) ^b^							
*syn*-DP	0.900	0.791	0.907		0.821	0.736	0.772
*anti*-DP	0.943	0.889	0.889		0.896	0.809	0.698
∑DPs	0.940	0.874	0.895		0.897	0.842	0.723

^a^ Correlation coefficients after controlling age and sampling day intervals among samples. ^b^ All correlations are significant at the 0.01 level (2-tailed).

## Data Availability

Not applicable.

## Supplementary Materials

The following supporting information can be downloaded at https://www.mdpi.com/article/10.3390/molecules27072242/s1, Table S1: Sampling time intervals between paired samples in different trimesters from the same participant. Table S2: Descriptive statistics for blood biochemical parameters in sequential blood samples. Table S3: Inter-period correlations, variation pattern, and variation extent (%) of POP levels across pregnancy in the present study.
