# Supplementary material for: Comparison of Dechlorane Plus Concentrations in Sequential Blood Samples of Pregnant Women in Taizhou, China
Source: Molecules. 2022 Mar 30;27(7):2242. doi: 10.3390/molecules27072242 (PMC9000586; doi:10.3390/molecules27072242)
Supplement: Supplementary file 1 [file molecules-27-02242-s001.zip › molecules-1607001-supplementary.pdf]

Supplementary Information for:

Comparison of dechlorane plus concentrations in sequential blood samples of pregnant women in Taizhou, China

Ji-Fang-Tong Li [a,b], Xing-Hong Li [a,b,*], Yao-Yuan Wan [a,b], Yuan-Yuan Li [a,b], Zhan-Fen Qin [a,b]

[a] State Key Laboratory of Environmental Chemistry and Ecotoxicology, Research Center for Eco-Environmental Sciences, Chinese Academy of Sciences, PO Box 2871, 18 Shuangqing Road, Haidian District, Beijing 100085, PR China

[b] University of the Chinese Academy of Sciences, 19A Yuquan Road, Shijingshan District, Beijing 100049, PR China

*Corresponding author: Xing-Hong Li

Tel: +86 10 62919177, fax: +86 10 62923563

E-mail: lxhzpb@rcees.ac.cn

1. Abbreviations:

PCP: Pentachlorophenol; DDT: Dichlorodiphenyltrichloroethane; HCB: Hexachlorobenzene; HCH: Hexacyclochlorohexane; DDE: Dichlorodiphenyldicloroethylene; PFOS: Perfluorooctanesulfonate; PFOA: Perfluorooctanoate; PFHxS: Perfluorohexanesulfonate; PFNA: Perfluorononanoate ; Me-PFOSA-AcOH: 2-(N-methylperfluorooctane sulfonamido) acetate.

2. Data analysis

In Table S3, variation extent (P, %) of POPs across pregnancy were evaluated from paired data in matched studies according to Eq.S1. C_1 represents the value of POPs levels measured in samples taken in earlier sampling period, C_2 represents value of POPs levels measured in samples taken in maximum sampling interval samples during pregnancy.

$$P = 100 \times (C_2 - C_1)/C_1 \cdots\cdots (S1)$$

3. Table S1. Information about the samples collection and the sampling time intervals between paired samples in different trimester from the same participants.

4. Table S2. Descriptive statistics for blood biochemical parameters in sequential blood samples.

5. Table S3. Inter-period correlations, variation pattern and variation extent (%) of POP levels across pregnancy in present study.

Table S1: Information about the samples collection and the sampling time intervals between paired samples in different trimester from the same participants.

Participant number	Samples collected during pregnancy[a]			Sampling time intervals/day		
	1st trimester	2nd trimester	3rd trimester	1st-2nd [b]	1st-3rd [c]	2nd-3rd [d]
1	1-1	1-2	1-3	81	137	56
2	2-1	2-2	2-3	108	196	88
3	3-1		3-3		178	
4		4-2	4-3			49
5	5-1	5-2	5-3	96	159	63
6	6-1	6-2	6-3	110	196	86
7	7-1	7-2	7-3	70	147	77
8	8-1		8-3		180	
9	9-1	9-2		98		
10		10-2	10-3			118
11		11-2	11-3			35
12	12-1	12-2	12-3	63	128	65
13	13-1	13-2	13-3	48	131	83
14	14-1	14-2		94		
15		15-2	15-3			67
16	16-1	16-2	16-3	76	146	70
17	17-1	17-2	17-3	112	154	42
18	18-1	18-2	18-3	84	161	77
19	19-1		19-3		128	
20	20-1		20-3		159	
21	21-1	21-2	21-3	119	141	81
22	22-1	22-2		114		
23	23-1	23-2	23-3	77	133	56
24	24-1	24-2	24-3	84	140	56
25	25-1	25-2	25-3	84	140	56
26	26-1	26-2	26-3	78	162	72
27	27-1	27-2		126		
28	28-1	28-2		107		
29	29-1	29-2		92		
30	30-1		30-3		146	

[a]: "m-n" represents sample from number m participant in number n trimester.

[b]: sampling time intervals between 1st and 2nd trimester.

[c]: sampling time intervals between 1st and 3rd trimester.

[d]: sampling time intervals between 2nd and 3rd trimester.

Table S2: Descriptive statistics for blood biochemical parameters in sequential blood samples.

Parameter	1st Trimester (<14 weeks)					2nd Trimester (14-28 weeks)					3rd Trimester (> 28weeks)					Reference interval[a]
	N	Median	Mean	SD	Range	N	Median	Mean	SD	Range	N	Median	Mean	SD	Range	
TC(mmol L^{-1})*	24	4.85	4.89	0.971	3.19-6.54	6	6.06	6.01	0.792	4.77-7.05	12	5.61	5.85	1.29	3.57-7.82	0.000-5.17
TG(mmol L^{-1}) *	24	1.10	1.25	0.466	0.670-2.16	6	2.11	2.11	0.645	1.27-3.19	12	2.41	2.65	1.17	1.19-4.88	0.000-1.69
HDL(mmol L^{-1})	23	1.83	1.81	0.371	1.07-2.78	5	2.01	1.96	0.177	1.69-2.16	12	1.83	1.85	0.349	1.39-2.49	1.03-3.00
LDL(mmol L^{-1})	23	2.72	2.63	0.699	1.37-3.84	5	3.51	3.28	0.685	2.15-3.90	12	2.98	2.96	0.719	1.60-4.05	0.000-3.36
Apo-A(mg dL^{-1})	23	170	173	39.9	92.0-264	5	197	190	16.2	166-205	12	170	171	32.1	122-236	100-200
Apo-B(mg dL^{-1})*	23	74.0	106	123	47.0-662	5	116	121	33.4	85.0-175	12	122	124	31.9	67.0-176	0.000-100
Lp(a) (mg dL^{-1})	23	48.3	45.7	10.3	27.7-60.7	5	30.4	27.0	13.9	4.40-41.5	12	17.3	18.3	10.6	0.000-37.9	0.000-30.0
Hb(g L^{-1}) *	23	128	136	44.7	108-338	19	117	112	12.4	80.0-125	21	112	105	26.9	3.80-132	120-160
ALB(g L^{-1}) *	23	43.8	43.4	8.99	21.3-74.9	23	38.7	38.3	2.56	32.8-44.1	20	36.5	34.7	10.4	11.8-64.7	35.0-55.0
Cre (μmol L^{-1})	22	46.2	46.1	6.02	33.3-55.4	14	45.0	45.5	3.88	37.4-49.9	19	49.4	46.8	11.6	3.50-58.6	44.0-106

*: Levels showed significantly difference between 1st trimester and 3rd trimester at p=0.05.

[a]: Reference interval for clinical biochemistry assays gained from hospital.

Table S3. Inter-period correlations, variation pattern and variation extent (%) of POP levels across pregnancy in present study.

	Wet-weight levels			Lipid-adjusted levels			STI(d)	N	Ref.
	Pattern	P, (%)	Correlation	Pattern	P, (%)	Correlation			
**Dechlorane plus**									
syn-DP	↑*	68	Y	↑	28	Y			
anti-DP	↑*	40	Y	↑	19	Y	196	20,20	Our study[d]
DP	↑*	41	Y	↑	20	Y			
**Organchlorine pesticides**									
Aldrin	↓	-	N	-	-	-	-	67,48	(Bloom et al. 2009)[b]
Mirex	↑	-	Y	-	-	-	-	67,48	(Bloom et al. 2009)[b]
PCP	↑	-48	N	-	-	-	-	8,8	(Glynn et al. 2011)[b]
p,p' -DDT	↑	1	-	-	-	-	180	14,14	(Roncevic et al. 1987)[c]
	↑	9	Y	↓	-26	Y	224	76,44	(Adetona et al. 2013)[a]
	↑*	100	-	-	-	-	280	45,39	(Junque et al. 2020)[c]
	↑#	300	-	-	-	-	280	39,101	(Takser et al. 2005)[b]
	↓#	-3	-	-	-	-	233	5,5	(Curley and Kimbrough 1969)[a]
o,p' -DDT	↑#	314	-	-	-	-	233	5,5	(Curley and Kimbrough 1969)[a]
p,p' -DDE	UN	0	Y	↑	1	Y	190	209,105	(Jarrell et al. 2005)[c]
	↑*	12	Y	↓	-2	Y	206	50,45	(Hansen et al. 2010)[c]
	↑	17	-	-	-	-	280	45,39	(Junque et al. 2020)[c]
	↑	23	-	-	-	-	180	14,14	(Roncevic et al. 1987)[c]
	↑#	24	-	-	-	-	280	39,101	(Takser et al. 2005)[b]
	↑#	35	Y	↓#	-17	Y	280	67,67	(Longnecker et al. 1999)[b]
	↑*	38	Y	↓*	-28	Y	224	76,44	(Adetona et al. 2013)[a]

	-	-	-	↑	35	-	280	203,203	(Wang et al. 2009)[c]
	↓	-	Y	-	-	-	-	67,48	(Bloom et al. 2009)[b]
	↑[#]	35	Y	↓[#]	-17	Y	280	67,67	(Longnecker et al. 1999)[b]
o,p' -DDE	↓[#]	-75	-	-	-	-	233	5,5	(Curley and Kimbrough 1969)[a]
HCB	↑	10	Y	↑	0	Y	190	209,105	(Jarrell et al. 2005)[c]
	↑[*]	10	Y	↓	-4	Y	206	50,50	(Hansen et al. 2010)[c]
	↑[*]	21	Y	↓[*]	-23	Y	224	58,55	(Adetona et al. 2013)[a]
	↑[#]	50	-	-	-	-	280	39,101	(Takser et al. 2005)[b]
	↑[*]	83	-	-	-	-	280	45,39	(Junque et al. 2020)[c]
	↓[*]	-	Y	-	-	-	-	67,48	(Bloom et al. 2009)[b]
α-HCH	↓	-3	-	-	-	-	280	45,39	(Junque et al. 2020)[c]
β-HCH	↑	3	-	-	-	-	180	14,14	(Roncevic et al. 1987)[c]
	↑[*]	85	-	-	-	-	280	45,39	(Junque et al. 2020)[c]
	↑	-	N	-	-	-	-	67,48	(Bloom et al. 2009)[b]
	-	-	-	↑	-29	-	280	203,203	(Wang et al. 2009)[c]
γ-HCH	↑	65	-	-	-	-	180	14,14	(Roncevic et al. 1987)[c]
	↓	-1	-	-	-	-	280	45,39	(Junque et al. 2020)[c]
cis-Nonachlor	↑[*]	25	Y	UN	0	Y	206	50,50	(Hansen et al. 2010)[c]
trans-Nonachlor	↑[*]	25	Y	↑	7	Y	206	50,50	(Hansen et al. 2010)[c]
	↑[#]	67	-	-	-	-	280	39,101	(Takser et al. 2005)[b]
	-	-	-	↑	22	-	280	203,203	(Wang et al. 2009)[c]
	↓[*]	-	N	-	-	-	-	67,48	(Bloom et al. 2009)[b]
Oxychlordane	↑[#]	50	-	-	-	-	280	39,101	(Takser et al. 2005)[b]
	↑[*]	-	N	-	-	-	-	67,48	(Bloom et al. 2009)[b]
**Polychlorinated biphenyls**									
PCB-52	↓	-11	-	-	-	-	280	45,39	(Junque et al. 2020)[c]

PCB-74	↑*	29	Y	↓*	-19	Y	224	76,59	(Adetona et al. 2013)[a]
PCB-99	↑*	36	Y	↓	-8	Y	224	76,59	(Adetona et al. 2013)[a]
	UN	0	Y	↓*	-10	Y	206	50,49	(Hansen et al. 2010)[c]
	UN#	0	-	-	-	-	280	39,101	(Takser et al. 2005)[b]
PCB-101	↑	9	-	-	-	-	280	45,39	(Junque et al. 2020)[c]
PCB-105	↑	19	Y	↓	-23	Y	224	76,59	(Adetona et al. 2013)[a]
PCB-118	↑	6	Y	↓*	-10	Y	206	48,49	(Hansen et al. 2010)[c]
	↑*	23	Y	↓*	-22	Y	224	76,59	(Adetona et al. 2013)[a]
	↑	33	Y	↓	-33	Y	196	7-10,7-10	(Glynn et al. 2011)[b]
	↑#	50	-	-	-	-	280	39,101	(Takser et al. 2005)[b]
	↓	-	Y	-	-	-	-	67,48	(Bloom et al. 2007)[b]
PCB-126	-	-	-	↑	29	-	280	203,203	(Wang et al. 2009)[c]
PCB-138	↑*	13	Y	↓	-2	Y	206	50,49	(Hansen et al. 2010)[c]
	↑#	33	-	-	-	-	280	39,101	(Takser et al. 2005)[b]
	↑	39	Y	↓*	-28	Y	196	7-10,7-10	(Glynn et al. 2011)[b]
	↑	74	-	-	-	-	280	45,39	(Junque et al. 2020)[c]
PCB-146	↑	9	Y	↓*	-32	Y	224	76,60	(Adetona et al. 2013)[a]
PCB-153	↑	8	Y	↑	5	Y	190	209,105	(Jarrell et al. 2005)[c]
	↑*	13	Y	↓	-2	Y	206	50,49	(Hansen et al. 2010)[c]
	↑*	21	Y	↓*	-23	Y	224	76,61	(Adetona et al. 2013)[a]
	↑#	29	-	-	-	-	280	39,101	(Takser et al. 2005)[b]
	↑	29	Y	↓*	-33	Y	196	7-10,7-10	(Glynn et al. 2011)[b]
	↑*	102	-	-	-	-	280	45,39	(Junque et al. 2020)[c]
	-	-	-	↑	8	-	280	203,203	(Wang et al. 2009)[c]
	↓	-	Y	-	-	-	-	67,48	(Bloom et al. 2007)[b]
PCB-156	UN#	0	-	-	-	-	280	39,101	(Takser et al. 2005)[b]

	↑*	13	Y	↓	-5	Y	206	47,49	(Hansen et al. 2010)[c]
	↑	100	Y	↓	-17	Y	196	7-10,7-10	(Glynn et al. 2011)[b]
PCB-163	↓	-10	Y	↓*	-23	Y	206	49,49	(Hansen et al. 2010)[c]
PCB-169	-	-	-	↑	21	-	280	203,203	(Wang et al. 2009)[c]
PCB-170	↑*	13	Y	UN	0	Y	206	46,48	(Hansen et al. 2010)[c]
	↑*	19	Y	↓*	-21	Y	224	76,62	(Adetona et al. 2013)[a]
	↑#	100	-	-	-	-	280	39,101	(Takser et al. 2005)[b]
PCB-180	↑*	12	Y	↓	-1	Y	206	50,48	(Hansen et al. 2010)[c]
	↑*	24	Y	↓*	-20	Y	224	76,63	(Adetona et al. 2013)[a]
	↑#	25	-	-	-	-	280	39,101	(Takser et al. 2005)[b]
	↑	35	Y	↓*	-37	Y	196	7-10,7-10	(Glynn et al. 2011)[b]
	↑*	75	-	-	-	-	280	45,39	(Junque et al. 2020)[c]
	-	-	-	↓	-1	-	280	203,203	(Wang et al. 2009)[c]
PCB-183	↑*	10	Y	↑	8	Y	206	49,47	(Hansen et al. 2010)[c]
	↑	14	Y	↓*	-28	Y	224	76,64	(Adetona et al. 2013)[a]
PCB-187	UN#	0	-	-	-	-	280	39,101	(Takser et al. 2005)[b]
	↑	6	Y	↓*	-33	Y	224	76,65	(Adetona et al. 2013)[a]
	↑*	13	Y	UN	0	Y	206	50,44	(Hansen et al. 2010)[c]
PCB-194	↑*	15	Y	↓	-6	Y	206	50,49	(Hansen et al. 2010)[c]
	↑*	22	Y	↓*	-15	Y	224	76,66	(Adetona et al. 2013)[a]
PCB-199	↑	14	Y	↓*	-28	Y	224	76,68	(Adetona et al. 2013)[a]
Total PCBs	↑#	18	-	-	-	-	280	39,101	(Takser et al. 2005)[b]
	↑	33	Y	↓*	-33	Y	196	7-10,7-10	(Glynn et al. 2011)[b]
	↑#	34	Y	↓#	-1	Y	280	67,67	(Longnecker et al. 1999)[b]
	↓*	-	Y	–	–	–	-	67,48	(Bloom et al. 2007)[b]
	↓	-11	Y	↓	-3	Y	190	209,105	(Jarrell et al. 2005)[c]

	↓	-1	-				180	14,14	(Roncevic et al. 1987)[c]
**Per- and polyfluorinated Compounds**									
PFOS	↓*	-11	Y	-	-	-	203	19,19	(Glynn et al. 2012)[b]
	↓*	-29	Y	-	-	-	175	71,71	(Kato et al. 2014)[c]
	↓*	-34	Y	-	-	-	245	48,48	(Fisher et al. 2016)[c]
	UN	0	-	-	-	-	56	44,38	(Fromme et al. 2010)[b]
PFOA	↓*	-16	Y	-	-	-	203	19,19	(Glynn et al. 2012)[b]
	↓	-21	-	-	-	-	56	44,38	(Fromme et al. 2010)[b]
	↓*	-30	Y	-	-	-	175	71,71	(Kato et al. 2014)[c]
	↓*	-38	Y	-	-	-	245	48,48	(Fisher et al. 2016)[c]
PFHxS	↓*	-29	Y	-	-	-	245	48,48	(Fisher et al. 2016)[c]
	UN	0	-	-	-	-	56	44,38	(Fromme et al. 2010)[b]
PFNA	↓*	-33	Y	-	-	-	203	19,19	(Glynn et al. 2012)[b]
	↓	-23	Y	-	-	-	175	71,71	(Kato et al. 2014)[c]
	UN	0	-	-	-	-	56	44,38	(Fromme et al. 2010)[b]
Me-PFOSA-AcOH	↓*	-50	Y				175	71,71	(Kato et al. 2014)[c]

1. STI: Sampling time interval(d), We calculated the longest sampling time intervals during pregnancy in matched literature.

2. N: number of samples, (m, n) represents m samples taken in earlier sampling period and n samples taken in the longest sampling time intervals during pregnancy.

3. *: Difference showed in inter-trimester POP levels at p=0.05 in matched literature.

4. #: No test of significance in matched literature, variation pattern gained from descriptive statistics.

5. -: No data and test of significance in matched literature; UN: Levels of POPs unchanged; Y means correlations between sequential samples were significant at p=0.05; N means correlations between sequential samples were not significant at p=0.05.

6. Because of the available data given in published literature, we calculate the variation extent in three statistical descriptions of available data, respectively. Details are as follow: **a**: Variation and correlation were calculated by Eq. S1, C values used mean levels of POPs in matched literature; **b**: Variation and correlation were calculated by Eq. S1, C values used median levels of POPs in

matched literature; **c**: Variation and correlation were calculated by Eq. S1, C values used geomean levels of POPs in matched literature; **d**: Extent calculated by Eq. S1, C values used median of P values by Eq. 2 in our study. Only two studies (Adetona et al. 2013),(Curley and Kimbrough 1969) use mean values and mean levels of POPs, that possibly contributed to the large variation extents.

References

Adetona, O., K. Horton, A. Sjodin, R. Jones, D. B. Hall, M. Aguillar-Villalobos, B. E. Cassidy, J. E. Vena, L. L. Needham and L. P. Naeher (2013). "Concentrations of select persistent organic pollutants across pregnancy trimesters in maternal and in cord serum in Trujillo, Peru." Chemosphere **91**(10): 1426-1433.https://doi.org/10.1016/j.chemosphere.2013.01.043.

Bloom, M. S., G. M. Buck-Louis, E. F. Schisterman, P. J. Kostyniak and J. E. Vena (2009). "Changes in maternal serum chlorinated pesticide concentrations across critical windows of human reproduction and development." Environ Res **109**(1): 93-100.https://doi.org/10.1016/j.envres.2008.08.013.

Bloom, M. S., G. M. Buck Louis, E. F. Schisterman, A. Liu and P. J. Kostyniak (2007). "Maternal serum polychlorinated biphenyl concentrations across critical windows of human development." Environ Health Perspect **115**(9): 1320-1324.https://doi.org/10.1289/ehp.10086.

Curley, A. and R. Kimbrough (1969). "Chlorinated Hydrocarbon Insecticides in Plasma and Milk of Pregnant and Lactating Women." Archives of Environmental Health: An International Journal **18**(2): 156-164.https://doi.org/10.1080/00039896.1969.10665387.

Fisher, M., T. E. Arbuckle, C. L. Liang, A. LeBlanc, E. Gaudreau, W. G. Foster, D. Haines, K. Davis and W. D. Fraser (2016). "Concentrations of persistent organic pollutants in maternal and cord blood from the maternal-infant research on environmental chemicals (MIREC) cohort study." Environ Health **15**(1): 59.https://doi.org/10.1186/s12940-016-0143-y.

Fromme, H., C. Mosch, M. Morovitz, I. Alba-Alejandre, S. Boehmer, M. Kiranoglu, F. Faber, I. Hannibal, O. Genzel-Boroviczeny, B. Koletzko and W. Volkel (2010). "Pre- and postnatal exposure to perfluorinated compounds (PFCs)." Environ Sci Technol **44**(18): 7123-7129.https://doi.org/10.1021/es101184f.

Glynn, A., U. Berger, A. Bignert, S. Ullah, M. Aune, S. Lignell and P. O. Darnerud (2012). "Perfluorinated alkyl acids in blood serum from primiparous women in Sweden: serial sampling during pregnancy and nursing, and temporal trends 1996-2010." Environ Sci Technol **46**(16): 9071-9079.https://doi.org/10.1021/es301168c.

Glynn, A., M. Larsdotter, M. Aune, P. O. Darnerud, R. Bjerselius and A. Bergman (2011). "Changes in serum concentrations of polychlorinated biphenyls (PCBs), hydroxylated PCB metabolites and pentachlorophenol during pregnancy." Chemosphere **83**(2): 144-151.https://doi.org/10.1016/j.chemosphere.2010.12.050.

Hansen, S., E. Nieboer, J. O. Odland, T. Wilsgaard, A. S. Veyhe and T. M. Sandanger (2010). "Levels of organochlorines and lipids across pregnancy, delivery and postpartum periods in women from Northern Norway." J Environ Monit **12**(11): 2128-2137.https://doi.org/10.1039/c0em00346h.

Jarrell, J., S. Chan, R. Hauser and H. Hu (2005). "Longitudinal assessment of PCBs and chlorinated pesticides in pregnant women from Western Canada." Environ Health **4**: 10.https://doi.org/10.1186/1476-069X-4-10.

Junque, E., S. Garcia, M. A. Martinez, J. Rovira, M. Schuhmacher and J. O. Grimalt (2020). "Changes of organochlorine compound concentrations in maternal serum during pregnancy and comparison to serum cord blood composition." Environ Res **182**: 108994.https://doi.org/10.1016/j.envres.2019.108994.

Kato, K., L. Y. Wong, A. Chen, C. Dunbar, G. M. Webster, B. P. Lanphear and A. M. Calafat (2014). "Changes in serum concentrations of maternal poly- and perfluoroalkyl substances over the course of pregnancy and predictors of exposure in a multiethnic cohort of Cincinnati, Ohio pregnant women during 2003-2006." Environ Sci Technol **48**(16): 9600-9608.https://doi.org/10.1021/es501811k.

Longnecker, M. P., M. A. Klebanoff, B. C. Gladen and H. W. Berendes (1999). "Serial levels of serum organochlorines during pregnancy and postpartum." Arch Environ Health **54**(2): 110-114.https://doi.org/10.1080/00039899909602244.

Roncevic, N., S. Pavkov, R. Galetinsmith, T. Vukavic, M. Vojinovic and M. Djordjevic (1987). "Serum Concentrations of Organochlorine Compounds during Pregnancy and the Newborn." Bulletin of Environmental Contamination and Toxicology **38**(1): 117-124.https://doi.org/Doi 10.1007/Bf01606568.

Takser, L., D. Mergler, M. Baldwin, S. de Grosbois, A. Smargiassi and J. Lafond (2005). "Thyroid hormones in pregnancy in relation to environmental exposure to organochlorine compounds and mercury." Environ Health Perspect **113**(8): 1039-1045.https://doi.org/10.1289/ehp.7685.

Wang, R. Y., R. B. Jain, A. F. Wolkin, C. H. Rubin and L. L. Needham (2009). "Serum concentrations of selected persistent organic pollutants in a sample of pregnant females and changes in their concentrations during gestation." Environ Health Perspect **117**(8): 1244-1249.https://doi.org/10.1289/ehp.0800105.
